# *Campanula lingulata* populations on Mt. Olympus, Greece: where’s the “abundant centre”?

**DOI:** 10.1186/s40709-016-0058-3

**Published:** 2017-01-14

**Authors:** Anastasia E. Tzortzaki, Despoina Vokou, John M. Halley

**Affiliations:** 1 Department of Biological Applications and Technology, University of Ioannina, Ioannina, Greece; 2 Department of Ecology, School of Biology, Aristotle University of Thessaloniki, Thessaloniki, Greece

**Keywords:** *Campanula lingulata*, Abundance patterns, Altitudinal gradient, Abundance centre hypothesis, Abundance–occupancy relationship, Multi-resolution fractal framework

## Abstract

**Background:**

The abundant-centre hypothesis (ACH) assumes that a species becomes more abundant at the centre of its range, where the environmental conditions are most favorable. As we move away from this centre, abundance and occupancy decline. Although this is obvious intuitively, efforts to confirm the hypothesis have often failed. We investigated the abundance patterns of *Campanula lingulata* across its altitudinal range on Mt. Olympus, Greece, in order to evaluate the “abundant centre” hypothesis along an elevation gradient. Furthermore, we explored the species’ presence and dynamics at multiple spatial scales.

**Methods:**

We recorded flowering individuals during the summer months of 2012 and 2013 along a series of transects defined by paths. We investigated whether the probability of acquiring a larger number of individuals is larger toward the centre of its altitudinal distribution. We also calculated mean presence and turnover at different spatial scales that ranged from quadrats of 10 × 10 m^2^ to about 10 × 10 km^2^.

**Results:**

We were able to identify an abundant centre but only for one of the years of sampling. During the second year, we noted a two-peak abundance pattern; with the first peak occurring at 650–750 m and the second at 1100–1300 m. Variability in the species-presence pattern is observed across a wide range of spatial scales. The pattern along the transect displays fractal characteristics, consistent with a dimension of 0.24–0.29. We found substantial changes of state between the 2 years at all resolutions.

**Conclusions:**

Our results do not contradict the ACH, but indicate that ecological distributions exhibit types of variability that make the detection of abundant centres more difficult than expected. When a random fractal disturbance is superimposed upon an abundant centre, we can expect a pattern in which the centre is difficult to discern from a single instance. A multi-resolution or fractal approach to environmental variability is a promising approach for describing this phenomenon.

## Background

An important issue for ecology is to understand species’ spatial distributions [1, 2]. However, even describing spatial patterns of species’ abundance and occupancy is a challenge for various reasons, including the fact that different aspects of these distributions are only manifest at specific scales [2–4]. As stated by Andrewartha and Birch [5], “Distribution and abundance are but the obverse and reverse aspects of the same problem”.

One well-documented, widespread pattern is a positive trend in a species’ occupancy–abundance (OA) relationship. Species that decline in abundance also tend to occupy fewer sites, while species that increase in abundance are more likely to be expanding their distribution [6–8]. Such positive relationships are well documented for various scales and habitat types [7] and hold for a variety of taxa including plants [9], butterflies [10, 11], fish [12–16] and birds [17–20]. Several mechanisms have been proposed to explain this type of relationship [21]. These may be classified as range-position, resource breadth, resource usage, and population dynamics [21]. Range-position mechanisms use the location of the study area relative to the overall species’ range. Resource-breadth explanations consider the effect of a variety of resources affecting abundance and distribution. Resource-usage explanations assume that species that are locally abundant and widespread also use resources that are locally abundant and widespread. Finally, population-dynamic explanations use patterns of growth or decline, local extinction and colonization in order to describe the observed OA pattern [21].

The abundant-centre hypothesis (ACH) proposes that a species is most abundant and prevalent at the centre of its range, where the environmental conditions are most favorable. The abundance distributions of species in space have long been associated with the concept of environmental gradients [5, 22–27]. When studying vegetation patterns, Whittaker [28–30], observed that the maximum abundances could vary at different places along any gradient but, in general, the abundances of most species declined relatively steadily as we move away from a maximum value. This maximum is often assumed to coincide, or be close to the point with the best conditions for that species in terms of environmental factors, such as moisture and temperature. Following Whittaker’s remarks, Brown [6] observed that the species with the highest local abundances also tend to inhabit a greater proportion of sites within that region and have wider geographic ranges. In other words, species are more abundant and occupy more space at the centre of their ranges where they find conditions more suitable for their survival. If, on average, abundances decline towards the edges of the species’ geographical ranges [31, 32] and species occupy a smaller proportion of an area when they are closer to the edges of their ranges, then positive occupancy–abundance relationships are likely to arise (see range position explanation) [21, 32–34].

In Brown’s formulation of the abundant-centre hypothesis (ACH) [6], local abundance reflects how well a particular site meets the needs of a species in the context of the multi-axis formulation of its niche. Axes include physiological characteristics (e.g. temperature tolerance) as well as ecological characteristics (e.g. response to predators or competitors). Brown assumed that because of spatial autocorrelation, sites close to each other would have broadly similar abilities to meet the many needs of a species and thus could form and exhibit a clear abundant centre. In this picture, moving away from this optimal centre decreases the chances of meeting all the needs of a species, and hence its population declines [6, 26].

The factors that are most commonly used to explain the main vegetation patterns around the world [35–45], are those that are readily available to us, such as climate or topographic parameters. In order for each to be used as a surrogate for determining “good” or “bad” conditions for a species to survive, there must be evidence that this variable does indeed correlate with more fundamental factors [46].

Air temperature, is considered a major determinant of the physiology, fitness and distribution of organisms. Thus, monitoring the response of organisms to spatial temperature variation across latitudinal or altitudinal variables is often used in order to understand temporal effects on the species’ local population dynamics [47–52]. In addition, altitudinal and latitudinal range shifts of plants as a response to global warming have been reported in several occasions [52, 53]. Altitudinal gradients are widely used as a study system—steep environmental gradients found on mountains provide us with the opportunity to explain the response of a species to gradual change of its environment over a short spatial distance [49]. Nevertheless, the differences in processes such as the spatial rate of temperature change that is higher in altitudinal gradients, and different levels of gene flow that may result in differing patterns of genetic differentiation and adaptation between the two spatial gradients, should be taken into account [52].

Distributions displaying an “abundant centre” (following the formulation of Brown above) have often been used as a basis for exploring ecological and evolutionary processes [26]. However, there had always been some concern about the fact that the ACH seemed to be accepted more on the basis of a theoretical need than after evidence from the field. The first systematic examination was in 2002, by Sagarin and Gaines [26]. After reviewing the literature, they found that the majority of species have abundance distributions that differ from the expectation of an “abundant centre”: only 39% of the direct tests supported the hypothesis. So, it seemed that the ACH could not meet the tests of empirical observation. These authors limited their analysis on empirical studies that focused on intra-specific variation over the entire geographical distribution of species. They did not examine studies on abundance distribution patterns over altitudinal gradients or local environmental clines. In addition, they did not provide an adequate explanation why the ACH mostly failed to hold up. A further review of empirical studies comparing central versus peripheral characteristics of plant populations for morphological and reproductive as well as demographic traits from Abeli et al. [54], yielded similar results to Sagarin and Gaines [26]. They concluded that the ACH is not strongly supported for plant demography, morphology or reproduction because it does not take into account the differences between and within taxa and ignores population history. Pironon et al. [55] evaluated the ACH by assessing three species’ performance in terms of genetics, physiology, morphology and demography against three centrality gradients (geographic, climatic and historical) and arrived to similar conclusions [55].

Our study system consists of a biennial plant of the Campanulaceae family, *Campanula lingulata* (native in Southeastern Europe and Turkey) on Mt. Olympus (2917 m), which is the highest mountain of Greece. The variable of interest is abundance; it is assumed to respond to elevation, which acts as a surrogate for the environmental predictors that shape the species distribution. We concentrate on part of a species distribution rather than on its full geographical range, assuming an analogy between latitudinal and altitudinal effects. We consider the distribution of a plant species along an altitudinal gradient and we investigate how well it complies with the ACH. In order to investigate whether there existed an underlying abundant centre pattern that is consistent with the observed distribution, we consider spatially explicit data.

While the ACH has been traditionally investigated at larger scales under the assumption that latitudinal gradients can be considered adequate surrogates for factors related to climatic variables such as air temperature that exhibits a direct physiological impact in living organisms, our study is investigating the ACH at a finer scale. We have good reason to believe that the species response will be the same since it is widely accepted that altitudinal gradients are similar to latitudinal gradients. Specifically, in fields such as climate change, there is an accepted direct correspondence [52]. In addition, earlier work on our study system [56–60] has confirmed the persistence of *C. lingulata* populations and a level of variation of reproductive, pollination and morphological/physiological traits within their specified altitudinal range.

We also use presence data in order to explore the patterns and the dynamics of the species’ mean presence and change of state for individuals. Since environmental factors make an impact on the distribution of plants at a range of different spatial scales, it makes sense that we conduct sampling and analysis of plant distributions at different scales of resolution. The fractal geometry approach [61, 62] assumes scale-symmetry, namely that the fractal dimension as well as other statistics of interest, is invariant to changes of scale. There is evidence that many environmental situations and phenomena (e.g. mountains, coastlines, rivers, clouds) have fractal properties [63–65] and that some individual species have approximately self-similar distributions across scales [61, 65, 66]. Kunin [61] argued that if distributions are fractal, scale-area curves should be linear, with a slope of 1 − D_b_/2 (where D_b_ is the box-counting dimension of the distribution). “As the fractal dimension measures the propensity of a pattern to fill space, the slope of a scale-area curve measures the degree to which a species’ population fills its geographical range. The steeper the slope, the sparser the distribution” [61]. The slope and height of a scale-area curve contain species-abundance information for a wide range of spatial scales thus giving a scale-independent description of abundance [61]. Accordingly, assuming that the species has a fractal distribution pattern [61, 62], the patterns of occupancy should be similar, regardless of the spatial scale in question. Thus, the distribution attributes should be scale independent. In such case, the same issues arising in latitudinal investigations of the ACH will also arise in our altitudinal gradient.

Finally, we expect that an understanding of the system selected for study and of the relevance of the ACH, is bound to provide some insight to the reasons why the ACH so often fails to hold.

## Results

**Fig. 1:**
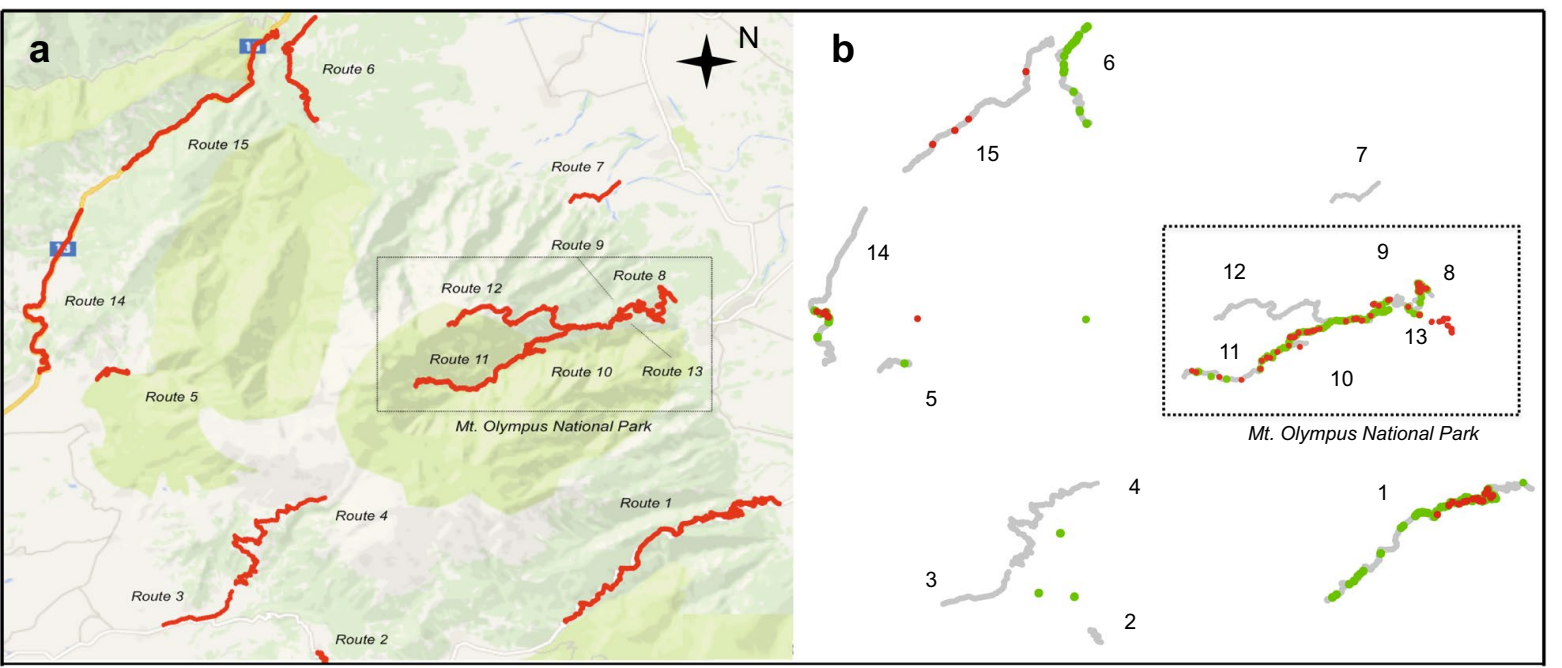
**a** Location of the routes (*decimal coordinates*) that were sampled within the overall study area of Mt. Olympus. The National park is found within the *dotted rectangle*. **b** Location of *C. lingulata* individuals (*decimal coordinates*) within the study area is marked in *red* for 2012 and *green* for 2013. Routes 2, 3, 4, and 7 were not included in the analysis since no individuals were observed. Singular observations outside the routes indicate confirmed presence of the species, but were not included in the analysis

**Fig. 2:**
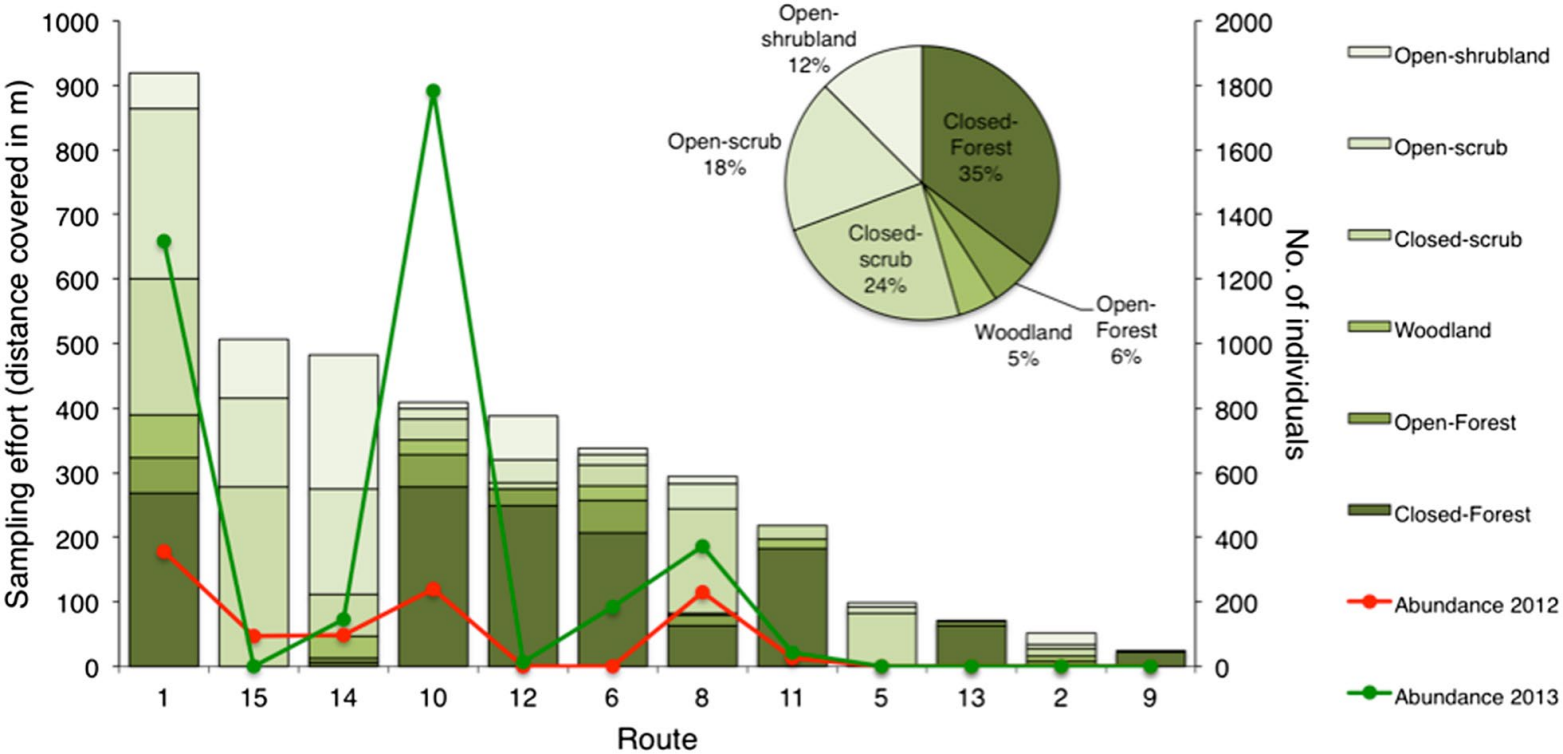
Invested sampling effort (no. of placemarks × 20 m) within each route and vegetation types per route; the latter are defined after the percentage foliage cover of the tallest plant layer. Given also is the percent sampling effort for each vegetation type after all routes (*pie chart*) and the total abundance of *C. lingulata* individuals for the year 2012 (*red line*) and 2013 (*green line*) per route. For details regarding placemarks, see “Methods” section

The location of the routes and the presence of *C. lingulata* individuals along them during the 2 years of study is shown in Fig. 1. Following the same routes on exactly the same periods, we recorded 1130 and 3897 individuals in 2012 and 2013, respectively (Fig. 2). Apart from the increase in abundance, which was observed in all but one route (15), Fig. 2 also shows the invested effort within each route as well as the percentage of each vegetation cover type of the categories described in the “Methods” section. The largest number of individuals was recorded in route 10, followed by routes 1 and 8.

**Fig. 3:**
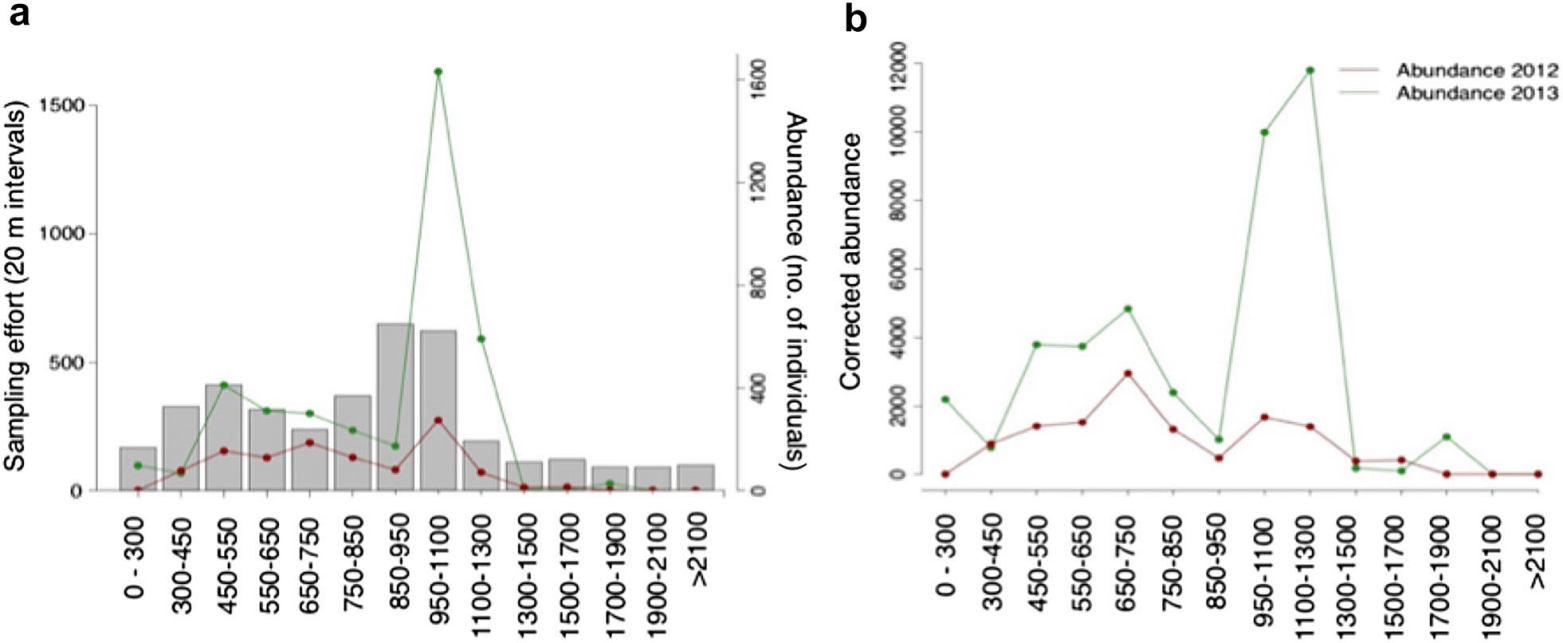
**a** Sampling effort per elevation class denoting the route length that was traversed within each elevation class (*bars*). If an observation was at the limits of each bin, it was included at the previous elevation class. Given also is the number of individuals in each elevation class for 2012 (*red line*) and for 2013 (*green line*). **b** Abundance corrected for invested effort for 2012 (*red line*) and 2013 (*green line*) for each elevation class

The number of individuals recorded in each elevation class is given in Fig. 3a. We observe a higher abundance around 950–1300 m in elevation for 2013, which could be considered towards the centre of the species altitudinal distribution, but in 2012, this distribution appears more or less homogeneous (Fig. 3).

Corrected abundance is shown in Fig. 3b. We note a two-peak pattern, with the first peak being around 650–750 m and the second around 1100–1300 m. This is followed by a sudden drop around 1300–1500 m; the latter may be attributed to the densely forested areas with no openings in routes 11 and 12 that include the highest altitudes (Fig. 2).

**Fig. 4:**
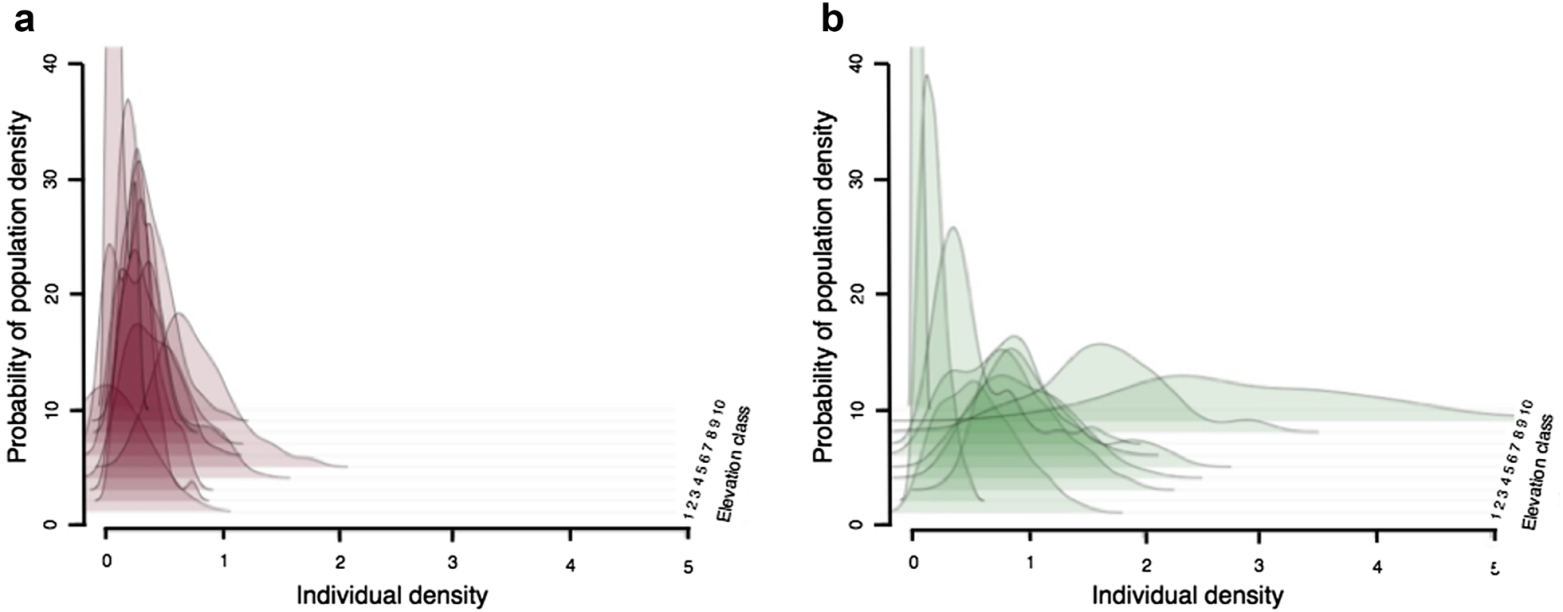
Probability density functions of the number of individuals in the altitudinal range 300–1300 m for **a** 2012 and **b** 2013. Each successive density curve corresponds to an elevation class. *X-axis* describes the density of individuals in a 20 × 20 m square, while *y-axis* denotes the probability of acquiring said density through random stratified sampling within each elevation zone. Numbers 1–10 for elevation classes are as described in Fig. 3

The probability density of the species abundance, expressed as the number of individuals in altitudes ranging from 350 to 1300 m, is displayed in Fig. 4a, b, for 2012 and 2013, respectively. Elevation classes above 1300 m are not featured since too few or no individuals were recorded. We cannot discern a notable increase in the probability of acquiring a larger number of individuals in any elevation class for 2012. In 2013, the probability of acquiring a larger number of individuals around 1100–1300 m is relatively greater, though in both cases most curves seem to overlap.

**Fig. 5:**
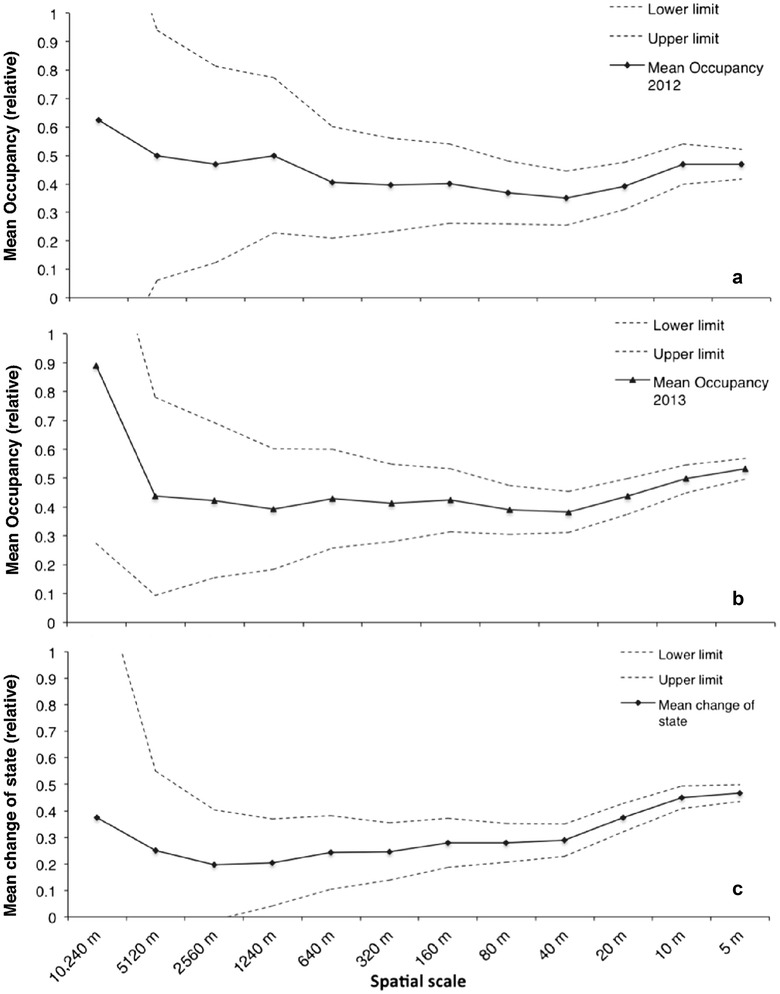
Mean relative occupancy (or occupancy at the intersection of the distribution with the transect) for **a** 2012 and **b** 2013. The upper and lower binomial proportion confidence intervals, assuming *p* follows a normal distribution for a = 0.05, are depicted as well. *X-axis* denotes the length of the cell side in m. **c** Mean change of state across the range of spatial resolutions.* Upper* and* lower* limits are as above

**Fig. 6:**
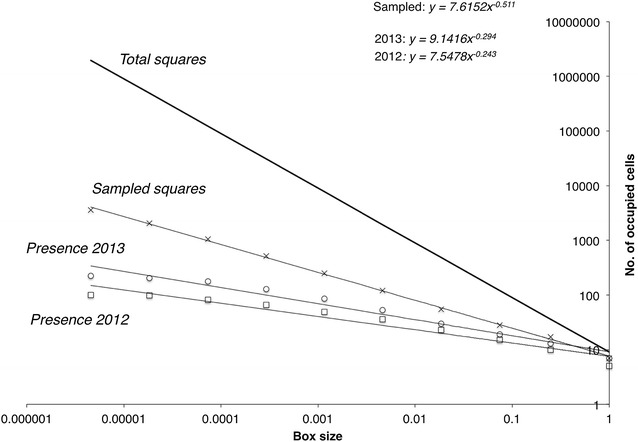
Occupancy of cells for *C. lingulata* in 2012 and 2013 along the intersection with the transect, number of total grid* squares*, and the corresponding cell numbers of the transect as a function of scale.* Box* counting fractal dimensions are the exponents in the displayed equations

Mean presence seems to remain constant for both 2012 and 2013 (Fig. 5a, b). This is consistent with a distribution with fractal properties (see Fig. 6). The species distributions for the 2 years of sampling have fractal dimensions 0.24 and 0.29, respectively.

*Campanula lingulata* flowers once, at the second year of its life cycle. For the purposes of this study, however, it is treated as annual. Regarding the mean change of state, we anticipate that at very coarse scales (as is the overall sampling area) there will not be any substantive change of state for occupied cells as the species is generally present in the area (mean population change of state close to 0) and it is not likely to change state. But at the scale of a single individual, it should be equal to 1, since each position at an individuals’ level cannot be occupied if it was occupied the year before (a given space cannot be occupied by flowering individuals for both successive years). We find that the observed turnover is larger than anticipated in the coarser resolutions, considering the spatial scales we discuss (Fig. 5c). The box-counting fractal dimension of the species distribution is given in Fig. 6.

## Discussion

Our initial hypothesis was that there would be some sort of bell-shaped distribution for *C. lingulata* along the altitudinal gradient on Mt. Olympus, with an abundant centre and a decline away from this towards zero, at the upper and lower limits. We find instead features that require explanation. We observe a single peak in overall abundance for 2012, while in 2013 we have a two-peak elevation pattern with an abrupt rather than smooth density decline at the upper limit (the 1300–1500 m elevation class).

The absence of a smooth distributional limit (Fig. 3a, b) can be understood in terms of the abrupt change in vegetation observed at higher elevations, whereas the striking double peak in abundance within the altitudinal range (Fig. 3) could be attributed to variations in habitat suitability. In the design of this study, altitude was perceived as a surrogate for climatic conditions. However, it also “summarizes” the effect of other factors and may be regarded as an approximation of the species’ multidimensional niche. Brown [6] described distributions of species that exhibit two or more peaks in abundance throughout space. According to his theory, this should occur when suitable habitat is found in isolated patches. Hence, the observed two-peak abundance pattern along the altitudinal gradient might be the combined result of altitude with gradients of other environmental factors that cannot be approximated by the gradual change in elevation, yet contribute to the formulation of the observed abundance patterns.

According to the ACH, we would expect higher probability of large abundances at the altitudinal “centre” of the species distribution. Our data support Sagarin and Gaynes [26] opinion, who concluded that the intuitive notion of an abundant centre is rarely upheld when put under empirical scrutiny. Our hypothesis of an abundant centre along an altitudinal gradient does not seem to hold, since we cannot observe the same pattern of abundance for both years of sampling (Fig. 4a, b). While the species appears most abundant at elevations that could be considered as the centre of its altitudinal range for 2013, the observed pattern in 2012 is rather homogeneous. Our results show that the ecological mechanism behind *C. lingulata* distribution patterns is not just the species’ optimal requirements regarding elevation. Nevertheless, the results do not contradict the ACH either.

The persistence of the abundant centre concept in the literature and its ubiquity in ecological and evolutionary theories reflect deeply held ideas by ecologists about how populations should be distributed. These are summarized in the abundant centre distribution pattern, which assumes underlying mechanisms and processes that are widespread in natural populations. However, more often than not, we fail to detect it [26]. Statistical approaches may adequately describe the observed distributions in space but they are rarely interpreted in a biologically meaningful context in respect to the underlying processes that shape species’ distributions. How then are we to interpret the observed data for occupancy and abundance if the patterns fail to connect a species’ distribution to the processes that shape it? Specifically, why do we not see the abundant centre which should have been there?

To answer this, we must interpret the abundance patterns that we observe along the environmental gradient in the context of other spatial and temporal features of the landscape and sampling design. The highest abundance is at the 1100–1300 m elevation class. Much of the effort invested in these elevations is within the National Park (routes 8 and 10), where human activities are regulated, as opposed to the total absence of individuals in routes 3 and 4, where intensive farming, grazing and other human activities take place. Grazing is reported to have a heavy impact on populations located outside the national park limits, but it does not affect high altitude populations [60]. The populations under investigation, however, (within and outside the park’s limits) are all close to footpaths or roads, so there are different levels of direct interventions that decrease with altitude (e.g. removal of the plants), and depend on whether the population is next to a road or a footpath.

Furthermore, our sampling is opportunistic. It reflects the availability of paths and road networks that cover the extent of our study area, thus introducing an error attributed to roadside bias. Our data did not permit extensive testing of whether the species’ abundance is correlated with the existence or absence of paths. Factors such as light exposure, which is greater in open areas as in roads, compared to densely forested areas, such as in routes 11 and 12, is related to the species presence or absence from certain elevation classes. Indeed, few to no individuals were recorded in densely forested areas of high elevation (Figs. 2, 3a, b). Thus, the elevational changes are heavily impacted by other factors.

Finally, it is important to note that only flowering individuals were recorded, with the duration of flowering reported to heavily depend on environmental conditions. According to Blionis et al. [57], flowering of individuals appeared to differ significantly both in terms of duration, and in terms of calendar days in 1992, which was a cold and wet year. May was cooler in 2012 than in 2013 (19.2 °C mean temperature, 107.4 mm total precipitation over 13 days, versus 21.4 °C and 118.2 mm total precipitation over 5 days for 2013) while June and July were hotter and drier in 2012 than in 2013 (25.6 °C, 9.6 mm over 2 days for June and 28.7 °C, 0.8 mm over 2 days for July 2012, versus 23.9 °C, 53.4 mm over 9 days for June and 26.3 °C, 39.4 mm over 9 days for July 2013). Temperature and precipitation data refer to the meteorological station of Dion Pierias [67]. The differences in abundance are bound to be an underestimate of the population number for 2012 since some populations may have flowered earlier or suffered severe losses due to the discrepancy in temperature and precipitation during the 2 years of study (Fig. 2).

The results in Fig. 5 indicate that the distribution of abundance of *C. lingulata* displays fractal properties as it does not seem to be scale dependent: mean presence of species individuals within each occupied square for each resolution remains rather constant across scales (Fig. 5a, b). We assume that the *C. lingulata* population of Mt. Olympus is a closed system, since the population is not likely to change state, unless a massive extinction event occurs. Under our assumption of a closed system, the species population turnover should have been close to 0. However, the estimated mean population turnover (Fig. 5c) is greater than anticipated at the broader spatial scales considered in this study. Such outcome may be indicative of a more dynamic system than expected.

Figure 6 shows that the species distributions for the 2 years of sampling have fractal dimensions 0.24 and 0.29, respectively. We consider the distribution along the path as being simply the intersection of the one-dimensional path with the fractal distribution. Using linear transects to sample multiscale distributions has always been a problematic issue but is necessary because we have limited sampling resources. When we are sampling fractal systems, the overall effect is well-known: our observed set retains a fractal character but with a lower dimension. Using a well-known intersection formula [Eq. (3) in Ref. 79], then we can infer the fractal dimension of the overall distribution itself on the mountain to be D = 1.24 or D = 1.29. However, it is unlikely that the distribution of the plant is independent of the path, since the path often provides unique conditions. For example, the open space and exposure to the sun might favor the species, relative to other areas and thus impact the observed patterns.

Theory and empirical evidence suggest that positive occupancy–abundance relationships result from the action of several mechanisms [21]. “Macroecological patterns are best understood as the net outcome of several processes pulling in the same direction [7, 21, 68, 69]”. Although several statistical OA and spatial distribution models have been proposed to quantify the observed OA patterns and explore the implications of such relationships, He et al. [70], in their review of OA models, argue that most of these fail to fully incorporate the effect of scale. Thus, while there is little doubt that multiple factors, operating across a hierarchy of spatial and temporal scales, shape species distributions [1], the lack of a theoretical framework connecting these scale-variant effects [71] means that little is known about how these determinants are connected across spatial scales [72–74].

Ecologists generally accept that broad scale processes constrain finer-scale phenomena. However, fine-scale processes (e.g. dispersal, various types of density dependence) may propagate to larger scales and impose constrains on the broad-scale patterns as well [75, 76]. In this context, a species’ distribution and its occupancy dynamics should be considered within a multi-resolution framework, such as the one that we have used here. It is evident that the mechanisms that shape occupancy–abundance patterns operate at various spatial scales. For a strictly fractal distribution, the slope encapsulates abundance information over all spatial scales into a single scale-independent description of abundance [61]. For more general distributions, this framework, though lacking a single slope and height to summarize all scales, it provides, nevertheless, a multi-resolution framework for exploring multi-scale processes behind species distributions.

## Methods

### The study system

Located on the border between Thessaly and Greek Macedonia, Mt. Olympus is the highest mountain in Greece (2917 m). At low elevations, the climate is typically Mediterranean (hot and dry in summer, while cold and rainy in winter). Vegetation can be distinguished into four zones: eu-mediterranean vegetation; beech, fir and mountainous para-mediterranean conifers; zone of cold-resistant conifers; and non-forested zone of high mountains [77, 78]. Mt. Olympus has been declared a National Park since 1938. The core of the park is located on the eastern side of the mountain, in an area of about 4000 hectares, whereas the peripheral zone of the National Park extends to about 24,000 hectares in total.

*Campanula lingulata* is a biennial hemicryptophyte. Its geographical distribution extends to Albania, Bosnia and Herzegovina, Bulgaria, Croatia, Greece, Italy, FYROM, Montenegro, Romania, Serbia, and Turkey [56]. It is the most common of the ten *Campanula* taxa on Mt. Olympus, where its altitudinal distribution extends from 200 to 1700 m [56]. *Campanula lingulata* is considered a pioneer species and its presence is favored in open habitats like those created under grazing pressure [60]. The genus has been thoroughly studied by Blionis [56], Blionis et al. [57], and Blionis and Vokou [58–60], who detected distributional, phenological, morphological, pollination and reproductive patterns for its representatives, and found a number of *Campanula* attributes to be strongly correlated with elevation [57, 58]. Pollination visitation rates to *Campanula* spp. flowers on Mt. Olympus decrease drastically with elevation and the composition of the pollinating fauna differs between lowland and upland populations [57, 58]. It flowers from late spring (mid-May) to early summer (early June) at lower elevations, and from early June to mid-/late July for middle to high elevations [56]. Duration of flowering is increasing with elevation to counterbalance the low number of seeds produced resulting from the low pollinator availability, and appears to vary in response to environmental conditions. *Campanula lingulata* populations from higher elevations also appear to have lower temperature optima for germination [60].

The level of human presence is reported by Blionis et al. [57] to cause reproductive losses either through grazing, or immediate human intervention. Grazing by domesticated animals during the summer period can have a severe impact on the short-lived populations of *C. lingulata*, ranging from 20% up to 90% losses, where populations do not reach fruit maturation and seed dispersal. The level of disturbance differentiates within the study area. Within the National park grazing is prohibited, and decreases with altitude outside park limits.

The existence of these earlier studies, primarily regarding its wide altitudinal distribution, sites of occurrence and flowering times, allowed us to consider *C. lingulata* as an ideal candidate for this study.

### Sampling

Sampling was carried out during the flowering season, since the plants are recognizable when in their flowering stage. Accordingly, the flowering season was divided in three sampling periods. The first covered low to middle elevations (200–1000 m); it started at mid-May and ended at mid-June. The second sampling period covered middle to high elevations (1000–1300 m); it started in mid-June and ended in mid-July. The last sampling period covered high elevations (1300–2500 m); it lasted from mid-July to late August. We carried out two full surveys, in 2012 and 2013. The second survey was a full repetition of the first; same routes were followed and same areas were visited. All *C. lingulata* individuals that were encountered on the mountain were recorded.

Our sampling was carried out along transects consisting of existing routes on Mt. Olympus that correspond to the road network and climbing paths. Selection of these routes was based on criteria of accessibility, positioning, directionality, length, elevation, habitat heterogeneity, and info (written and oral) on presence of *Campanula* species. In total, we surveyed 15 routes. Four of these, in the southwest side of the mountain, were not analysed due to complete absence of *C. lingulata* individuals. The total length surveyed was 74 km.

As illustrated in Fig. 1, route 6 is at the north side of the mountain, routes 2 and 5 at the southern, routes 14 and 15 at the northwest, and route 1 at the southeastern side. Routes 8, 9, 10 and 13 are located inside the National Park at the northeastern side of the mountain, and alongside the river Enipeas; of the latter, route 13 is densely forested and very close to the river’s bank. Route 11 and 12 are paths also within the National park, away from the river; route 11 is a densely forested path, while route 12 reaches the highest vegetation zone. Routes 2, 3, 4 and 7 are not included in the analysis. Human activity is rather intense in routes 2 and 5 and to a less degree in route 1 and also in routes 14 and 15, which are along a motorway; route 14 also traverses a populated area.

We recorded all individuals in bloom, within a band of 20 m on either side of the route, and estimated their position using a hand-held GPS device (eTrex Vista HCx, Garmin International). To facilitate calculations, we stored their coordinates in decimal degrees. We noted the vegetation of the surroundings in 20 m intervals, along each sampled route, and assigned them to categories based on the percentage foliage cover of the tallest plant layer, as observed in Google Earth (see *placemarks* below) [79] (Fig. 2). The categories were: closed forest (70–100% cover), open forest (30–70%), woodland (10–30%), closed-scrub (70–100%), open-scrub (30–70%), and open shrubland (<10%).

### Mapping the species in the surveyed area at different spatial resolutions

We defined a quadrangle on the Earth’s surface as the study area (Fig. 1). Within this quadrangle we produced nested grids of varying resolution that overlay the study area. For the coarsest partition the study area was initially divided by a 3 × 3 grid. Thereafter, at each partition, each cell was divided into four (2 × 2) sub-cells. This was repeated ten times, so the finest grid contained 3 × 2^10^ = 3072 columns of side 10 m and the same number of rows (9,437,184 cells in total). Thus we have twelve nested grids of varying resolutions that overlay the surveyed area and assigned each observation to its corresponding position (Table 1). Each observation could then be assigned to one cell within each grid depending on its position. Thus, our observations are contained in eleven matrices, ranging from fine (10 × 10 m) to coarse (10,240 × 10,240 m) resolution. The majority of the cells in finer resolutions were not surveyed, as observations were carried out only along certain routes. Thus, each grid cell is tagged with an ID value only if it contains observation*s*. An observation is either zero, indicating that no individuals were observed there, or a positive value corresponding to the number of individuals observed in that cell. The cells that did not intersect the transects (routes) at any spatial resolution, were considered not sampled.

**Table 1:** Cell numbers, cell-side lengths, and longitude and latitude intervals for each resolution

Scale ID (k)	Columns (or rows) (N)	Cell-side length, s_k_, (m)	Longitude interval (decimal degrees)	Latitude interval (decimal degrees)	Cell area (m^2^)
0	1	30,720	–	–	943,718,400
1	3	10,240	0.12288	0.09216	104,857,600
2	6	5120	0.06144	0.04608	26,214,400
3	12	2560	0.03072	0.02304	6,563,600
4	24	1240	0.01536	0.01152	1,537,600
5	48	640	0.00768	0.00576	409,600
6	96	320	0.00384	0.00288	102,400
7	192	160	0.00192	0.00144	25,600
8	384	80	0.00096	0.00072	6400
9	768	40	0.00048	0.00036	1600
10	1536	20	0.00024	0.00018	400
11	3072	10	0.00012	0.00009	100

### Mean population density estimate per elevation class

In order to determine the relative population abundance (or density in a 20 × 20 m square) in each elevation class, we had to correct for the fact that sampled cells are not equally distributed between elevation classes. In order to determine survey effort invested in each elevation class, each sampled route was marked with placemarks. Each placemark corresponds to a set of coordinates for longitude, latitude, elevation with respect to sea level and vegetation density (acquired from Google Earth). The first placemark in each route corresponds to each sampling route’s starting point, whereas the next was placed 20 m further, following the route in Google Earth (Fig. 1). To account for differences in effort invested in different elevations, the altitudinal range was divided into 14 elevation classes. Below 2100 m, the range was divided in 13 classes of 100 to 200 m change in altitude, whereas above it, all values were included in one class. Each placemark was assigned to an elevation class according to its elevation from the sea level. Finally, each placemark was placed in a 1536 × 1536 (20 m × 20 m) matrix. Each value ID that corresponded to a cell containing a placemark was considered sampled. Thus, each sampled square in this grid has an ID tag that corresponds to its elevation class and abundance, which is the number of individuals observed in that square. Effort was defined as the number of 20-m length intervals within each elevation class. The correction for abundance relative to sampling effort with elevation was calculated as:

1 $$n_{i} = \frac{{N_{i} L}}{{l_{i} }}$$

where *i* is the elevation class, *N*_*i*_ is the overall number of individuals for elevation class *i*, *L* is a measure of the overall sampling effort and refers to the total length (in 20-m intervals) of every route, and *l*_*i*_ the number of placemarks at each elevation class quantifies the effort invested in each elevation class.

Finally, we calculated the mean population density for 2012 and 2013, as the mean value of 100 sets of 100 randomly selected sampled squares per elevation class, drawn from a 20 × 20 m resolution grid. Their probability density function, which is the probability of acquiring a given number of individuals in each elevation class, was estimated with Gaussian kernel density estimation as a smoothing function.

### Multiresolution statistics: mean relative occupancy, change of state and box dimension

An important description of the spatial distribution is how occupancy changes between different scales and different years. The relative mean occupancy *p*_*k*_ is defined for a given year (t = 2012, 2013) at a given spatial resolution *k* (0 ≤ *k* ≤ 11).

Consider the numbers of occupied squares intersecting with the transect in two successive resolutions (spatial scales). An observation that is located within a cell at resolution *k* may be located in any of the four different sub-squares in the next (finer) scale relative to its centre, since each square is comprised by 2 × 2 cells in the finer resolution. Suppose that, in the coarse resolution *k*, *n*_*k*_ squares are occupied in the entire grid. Suppose that, in the finer resolution *k* + 1, *n*_*k*+*1*_ squares are occupied. We define the relative mean occupancy *p*_*k*_ as:

2 $$p_{k} = \frac{{n_{{k + {\mathbf{1}}}} }}{{{\mathbf{4}}n_{k} }},\;\;\;\;\;\;\;\;\;\;{\mathbf{k}} \in {\mathbf{0,1,}} \ldots, {\mathbf{10}}$$

Since each cell can be divided into four sub-cells, one at least of which must be occupied, it is clear that *p*_*k*+1_ must lie between 1 and ¼. The one exception is *p*_0_, since there are nine sub-cells for the first level, thus *p*_0_ = *n*_1_/*n*_0_ = *n*_1_/9 (since occupancy overall is equal to 1). Each square we find occupied is considered observed at a given resolution. If a sub-square is not occupied, we exclude it when we calculate relative mean occupancy for the next resolution.

We estimated the mean change of state *Δn*_*k*_ as the proportion of the sub-squares intersecting with the transect that have changed state between the 2 years of sampling, divided by the number of squares that were occupied in both years (sum of squares that were occupied both in 2012 and 2013, and squares that were occupied either only in 2012 or 2013) at a given resolution. Unoccupied squares that were not sampled at the coarser resolutions were excluded stepwise from the calculations. So, if *x*_*kj*_ is the occupancy of square *j* at the *k* ^*th*^ grid (either 0 or 1) then the mean change of state is:

3 $$\Delta n_{k} = \frac{{\sum\nolimits_{j} {\left| {x_{kj} (2013) - x_{kj} (2012)} \right|} }}{{\sum\nolimits_{j} {\hbox{max} \left( {x_{kj} (2013),x_{kj} (2012)} \right)} }}$$

Here, a numerator term is only 1 if there is a change of state between the 2 years while in the denominator a term is only 0 if *x* is zero in both years.

For the box dimension (or box-counting dimension), using the set of nested regular grids, we use the following procedure [62]:

i. Overlay the first grid onto the set of point data.
ii. Note the box-width, *s*_*k*_ and count the number of occupied boxes, *N*_*k*_.
iii. Repeat steps (i) and (ii), for each of the nested grids.
iv. Plot the logarithm of box numbers log[*N*_*k*_] as a function log(1/*s*_*k*_).
v. The slope of this log–log graph is an estimate of the box-counting dimension.

Data processing was performed in R statistical and programming environment, version 3.13 [80].

## Conclusions

The failure of generations of empirical studies to support our intuitive notions of the mechanisms generating the species abundance distributions calls for a revision of our conceptual framework. Any new framework must incorporate the effect of scale. We hypothesize that the combined effect of different, scale-variant factors is bound to superimpose the effect of biological drivers that could provide us with evidence of an underlying “abundant centre” species distribution. A multi-resolution or fractal framework, wherein the factor “scale” is inherent, might constitute a suitable approach.

In theory, we could always discern a species’ distribution centre, where it meets its optimal conditions, if we had enough instances (replicates) for different years. However, simple replication is hard to obtain for *C. lingulata* on Mt. Olympus because we cannot simply replicate the conditions, which are a result of disturbances on multiple spatio-temporal scales [81, 82]. Also, there is no clear line between the regular conditions and environmental variability or “noise” since this happens on many different scales. Hence, it is not possible to construct a statistically valid sample for statistical analysis in the usual sense. Simulations have proven to be valuable tools in such instances [83, 84]. An appropriate framework to investigate the ACH would be a spatially explicit multi-resolution model of disturbance, where each position within the species’ geographic range has a probability of occurrence based on its distance from the hypothetical centre and on noise from a multi-scale model of disturbance. Although beyond the intended scope of this study, such an approach provides an attractive direction for future work.

## Abbreviations

ACH: abundant centre hypothesis
OA: occupancy–abundance relationship
